# Effect of Fibrin Glue on the Biomechanical Properties of Human Descemet's Membrane

**DOI:** 10.1371/journal.pone.0037456

**Published:** 2012-05-25

**Authors:** Shyam S. Chaurasia, Ravi Champakalakshmi, Ang Li, Rebekah Poh, Xiao Wei Tan, Rajamani Lakshminarayanan, Chwee T. Lim, Donald T. Tan, Jodhbir S. Mehta

**Affiliations:** 1 Tissue Engineering and Stem Cell Group, Singapore Eye Research Institute, Singapore, Singapore; 2 Department of Bioengineering and Department of Mechanical Engineering, National University of Singapore, Singapore, Singapore; 3 Mechanobiology Institute, Singapore, Singapore; 4 Singapore National Eye Centre, Singapore, Singapore; 5 Department of Ophthalmology, Yong Loo Lin School of Medicine, National University of Singapore, Singapore, Singapore; 6 Department of Clinical Sciences, Duke-NUS Graduate Medical School, Singapore, Singapore; University of Missouri-Columbia, United States of America

## Abstract

**Background:**

Corneal transplantation has rapidly evolved from full-thickness penetrating keratoplasty (PK) to selective tissue corneal transplantation, where only the diseased portions of the patient's corneal tissue are replaced with healthy donor tissue. Descemet's membrane endothelial keratoplasty (DMEK) performed in patients with corneal endothelial dysfunction is one such example where only a single layer of endothelial cells with its basement membrane (10–15 µm in thickness), Descemet's membrane (DM) is replaced. It is challenging to replace this membrane due to its intrinsic property to roll in an aqueous environment. The main objective of this study was to determine the effects of fibrin glue (FG) on the biomechanical properties of DM using atomic force microscopy (AFM) and relates these properties to membrane folding propensity.

**Methodology/Principal Findings:**

Fibrin glue was sprayed using the EasySpray applicator system, and the biomechanical properties of human DM were determined by AFM. We studied the changes in the “rolling up” tendency of DM by examining the changes in the elasticity and flexural rigidity after the application of FG. Surface topography was assessed using scanning electron microscopy (SEM) and AFM imaging. Treatment with FG not only stabilized and stiffened DM but also led to a significant increase in hysteresis of the glue-treated membrane. In addition, flexural or bending rigidity values also increased in FG-treated membranes.

**Conclusions/Significance:**

Our results suggest that fibrin glue provides rigidity to the DM/endothelial cell complex that may aid in subsequent manipulation by maintaining tissue integrity.

## Introduction

Corneal endothelial dysfunction accounts for the majority of corneal transplantations performed worldwide. In the United States alone, corneal transplantation for diseased corneal endothelium, such as aphakic or pseudophakic bullous keratopathy and Fuchs' endothelial dystrophy, accounts for over one-third of all cases of corneal transplantations [1]–[3].

Advances in corneal transplantation over the last decade have led to the possibility of selectively replacing the corneal endothelium without the need of full thickness tissue replacement. Since the description by Melles et al. of posterior lamellar keratoplasty (PLK) in 1998 [4], endothelial keratoplasty (EK) has evolved through various iterations to the currently popular techniques of Descemet's stripping automated endothelial keratoplasty (DSAEK) and, more recently, Descemet's membrane endothelial keratoplasty (DMEK) [5]–[10]. In DSAEK, the donor endothelial cell layer is transplanted together with a thin layer of accompanying stromal tissue (100–200 µm) that acts as a scaffold and confers some structural rigidity to the donor tissue. This structural element allows the delicate donor tissue to be manipulated and delivered into the anterior chamber of the eye, where the graft is subsequently attached to the posterior surface of the host cornea by an air bubble [10]–[11]. DSAEK has faster and better long-term visual results compared to PK [11]–[13]. Even though there is significant improvement in results with DSAEK compared to PK, there is still the presence of a stromal-to-stromal optical interface that could potentially degrade visual recovery [11], [14]. DSAEK also causes an initial hyperopic refractive shift associated with the meniscal shape of the transplant on the posterior corneal curvature [12], [15].

Descemet's membrane (DM), also known as the posterior limiting lamina or membrane of Demours, is a basement membrane that lies in-between the stroma and the endothelial layer of the cornea [16], [17]. DM is composed of a highly elastic collagenous structure organized into a three-dimensional filamentous network. The thickness of DM increases with age, from 4 µm to 10–15 µm [18], [19]. The development of DMEK allows surgeons to selectively transplant endothelial cells and DM, resulting in rapid post-operative visual recovery, without significant refractive changes [8]–[10], [20]. Despite these advantages, the widespread acceptance of DMEK has been hindered by considerable difficulties in terms of tissue manipulation during surgery. The absence of a thin stromal scaffold in DMEK results in an extremely delicate tissue that has a natural tendency to scroll and tear easily during surgical manipulation.

Fibrin glue (FG), a biological tissue adhesive, has been widely used in several ophthalmic applications as a structural filler or support to tissue in the treatment of corneal perforations, conjunctival graft surgery, sutureless lamellar keratoplasty and leaking blebs during glaucoma surgery [21]–[24]. Fibrin glue has also been used extensively for treating corneal perforations through multilayered amniotic membrane transplantation [25]. Although the tensile strength of FG is not as strong as sutures, its ability to cause minimal inflammation and biodegradability make it an excellent candidate for a number of surgical applications [26], [27]. Recently, FG has also been modified with other natural and synthetic polymers such as gelatin, chondroitin-6-sulphate and polyvinyl-alcohol covinylamine to increase its adhesive nature [26], [28]. Fibrin glue has also been used as a hydrogel scaffold in ophthalmology [29], [30].

We hypothesize that FG applied to the endothelial graft during DMEK surgery may provide a temporary rigid scaffold to support the structural integrity of the donor tissue for easier delivery and manipulation of graft within the anterior chamber. The purpose of this study was to evaluate the biomechanical properties of DM coated with and without FG derived from nanoindentation and flexibility tests performed using atomic force microscopy (AFM). A surface topographical analysis of DM with and without FG was performed using scanning electron microscopy (SEM) and AFM.

## Materials and Methods

### Ethics statement

The present study conformed to the tenets of the Declaration of Helsinki and was approved by the institutional review board of Singapore National Eye Centre, Singapore.

### Descemet's membrane (DM) preparation

Human corneas stored in OPTISOL-GS (Bausch & Lomb Inc. NY, USA) were obtained from Lion's Eye Bank (Miami, FL, USA) [mean age = 56±16 years (range = 28–73 years); death to tissue harvest time = 12.5±7 hrs (range = 2–20 hrs); mean death to experiment time = 16±2 days (range = 13–18 days)]. All the surgeries were performed by JSM. The corneoscleral rims were washed in antibiotic/antimycotic solution for 15 minutes. The DM/endothelial sheets were isolated using a modification of previously described stripping method [31], [32]. Briefly, the corneoscleral rims were placed, endothelial side up on a disposable coronet corneal graft vacuum donor punch (Network Medical Products, North Yorkshire, UK) and stabilized by creation of vacuum suction. The DM was gently scored with blunt forceps circumferential at the level of Schwalbe's line. A 8.5 mm demarcation line was made with a corneal punch trephine ensuring perforation of DM/endothelial only. The corneoscleral rim was immersed in trypan blue solution (0.2%) for thirty seconds to improve visualization and maneuvering during the separation process. The 8.5 mm demarcated DM-endothelial layer was then carefully stripped off using a two fine forceps from the posterior stroma under a dissecting microscope (Nikon SMZ1500, Kanagawa, Japan).

### Fibrin glue (FG) preparation

The FG was reconstituted according to the manufacturer's protocol (TISSEEL VH Fibrin Sealant, Baxter Healthcare (Asia) Pte Ltd, Singapore). The kit consists of TISSEEL and thrombin serving as two major components of the FG. The Tisseel powder was dissolved in Aprotonin solution and stirred gently on the FIBRINOTHERM device (Baxter Healthcare) at 37°C until complete dissolution. The thrombin solution was prepared in CaCl_2_ and stirred on the FIBRINOTHERM. 20 µl of 0.5% trypan blue was added to 2 ml of Thrombin-CaCl_2_ solution to allow visualization of the spread of FG after spray. Finally, an EasySpray applicator (Baxter Healthcare, Singapore) that operates with the dual syringe system was used to draw the separate components of the FG. A spray head was fastened to the syringe system with a plunger and CO_2_ gas regulator, which regulated the flow of FG.

### Histology

The DM sprayed with and without the FG preparation was embedded in OCT and 8 µm fresh-frozen sections were obtained using a cryostat (Carl Zeiss MicroImaging GmbH, Jena, Germany). The sections were stained with hematoxylin and eosin to examine histology and evaluate the thickness after FG application under an Axioplan, Zeiss Light Microscope (Carl Zeiss MicroImaging) in bright field mode.

### Sample preparation for AFM measurements

**Figure 1:**
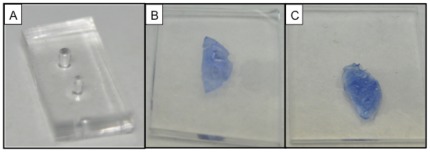
PDMS gel substrate for AFM nanoindentation. PDMS gel punched with 1.2 mm and 1.5 mm holes with punching pens (A); one half of the harvested human Descemet's membrane (DM) was placed on the punched holes (B), and the other half sprayed with fibrin glue (FG) was placed on the gel, covering the punched holes with the glue side facing down (C) for comparison of the biomechanical properties of DM with and without FG.

**Figure 2:**
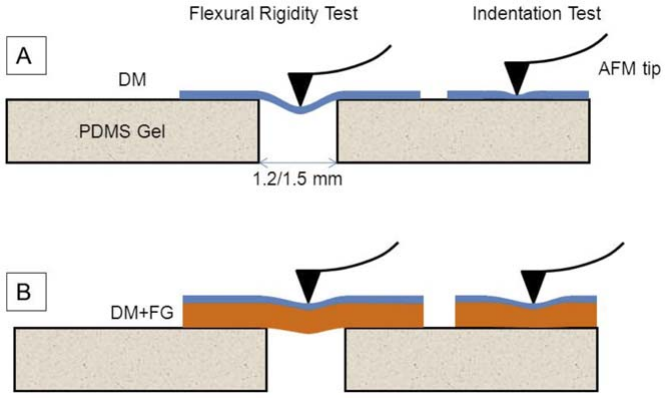
Schematic diagram of a Descemet's membrane (DM) with and without fibrin glue (FG) used for nanoindentation by atomic force microscopy (AFM). Indentation tests were performed on DM mounted on PDMS substrate while flexural tests were performed on DM mounted on PDMS substrate circular holes of diameters 1.2 mm or 1.5 mm. (A) shows a setup for a native DM and (B) shows the DM sprayed with FG with the glue facing downwards and attached to PDMS substrate.

The mechanical properties of DM were measured using a Dimension Icon AFM equipped with Nanoscope V controller (Bruker Corp., Santa Barbara, CA, USA). DM measuring 8.5 mm in diameter was placed on a polydimethylsiloxane (PDMS) substrate punched with 1.2 mm and 1.5 mm holes using harris unicore punch (Tedpella Inc., Redding, CA) (Fig. 1A–C). PDMS gel was prepared using SYLGARD 184 gel kit, which contained two components, silicone elastomer base and a curing agent. The components were mixed thoroughly in the ratio of 1∶10 and poured into a petriplate. This mixture was degassed for 1 hr in a desiccator and allowed to cure for 2 hrs in a hot air oven at 80°C. The gel was cut into slices of squares measuring 12 mm thick and 2 cm long to serve as a substrate for further studies. Each DM sample (n = 3) was cut into 2 semicircles; one half was used as a control and the other half was sprayed with FG. It was sprayed on the DM at a previously optimized distance of 5 cm and pressure of 20 psi to operate in a fast setting time of 1–2 minutes with an EasySpray applicator system as described earlier [33]. The native DM was placed on the punched holes and allowed to dry. The DM sample with FG was flipped over so that the DM side was facing upwards for the measurements. The schematic representation of the experimental setup is shown in Figure 2.

### AFM nanoindentation

The nanoindentation measurements were performed using a silicon TESPA cantilever (Bruker Corp., Santa Barbara, CA) with a V-shaped tip of 5–10 nm radius with a spring constant of the tip ranging from 55–80 N/m calibrated for each experiment. The tips were decontaminated by exposing to UV light for 10–15 minutes prior to testing. The DM samples were kept in semi-dry conditions throughout the experiment. The local slopes of all the three test specimens (DM, DM sprayed with fibrin glue (DM+FG) and fibrin glue (FG) only) were compared using force curves obtained from AFM tip indenting the specimens placed on a flat PDMS substrate. Force curves were also obtained for the flexibility tests performed by indenting on the center of DM placed exactly over 1.2 mm and 1.5 mm holes (Figs. 2A and 2B). Altogether, ∼10–15 force curves obtained for each measurement/location within indentation depth range of 200–500 nm and were compared between the control and test samples. Data was collected separately from three control (DM) samples age matched with three test samples (DM+FG) and FG for each experiment. Hence, there was no variation in the age of the donor between controls and samples.

#### Comparison of local slopes obtained from the force curves among different treatment groups

The force curves obtained were used to compare the local slopes of all the three test specimens (DM, DM+FG and FG). The parameters obtained from the force curves were piezo displacement (z) and cantilever deflection (d) in nanometers. The indentation depth was obtained from the difference between piezo displacement (z) and deflection (d) (δ = z – d). Relative values of indentation depth were calculated from the contact point (z_o_, d_o_) where the tip first contacted the sample surface.

The force (F) vs. indentation depth (δ) curves was first plotted from data obtained for DM, FG and DM+FG. The curves were then fitted with the following relation:
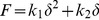
(1)where F is the loading force of the AFM cantilever tip, k is a constant and δ is the indentation depth. Equation 1 was further differentiated to find the local slopes of the curves as:
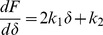
(2)Plots between dF/dδ vs. δ would then give the comparison between the slopes of DM, DM+FG and FG at different indentation depths.

#### Hysteresis measurements

Hysteresis measured in terms of energy loss during the loading of force (indentation) and unloading (retraction) was calculated from the loading and unloading force vs. indentation and retraction force curves. The area under each force curves, for indentation (A_i_) and retraction (A_r_), was calculated by summing up the areas of the trapeziums formed under each curve. Hysteresis was calculated by subtracting the area of retraction (A_i_) from the area of indentation (A_r_). The relative hysteresis was calculated by dividing the hysteresis values with the area of indentation.

#### Flexural rigidity measurements

The force curves obtained from the application of a point load on the center of a fully supported DM mounted over a circular hole (on the PDMS substrate) were analyzed [34]. We used the equation:
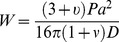
(3)where W is the deflection of DM at the center; ν is the Poisson's ratio (0.5 assuming that the biological membrane is incompressible); P is the force applied and D is the flexural or bending rigidity. Indentation depth in the range of 200–500 nm was compared between the samples with or without FG.

### Scanning Electron Microscopy (SEM)

Following AFM nanoindentation analysis, the samples were immediately fixed in 2% cold glutaraldehyde, 2% paraformaldehyde and 0.1 M sodium cacodylate buffer, pH 7.4 (Electron Microscopy Sciences, WA, USA) for overnight at 4°C. The tissues were then washed in buffer and secondarily fixed in 1% osmium tetroxide (Electron Microscopy Sciences). The samples were dehydrated, subjected to critical point drying and mounted on SEM stubs. They were sputter-coated with 10 nm of gold and examined with a SEM (XL30 FEG SEM; FEI Company/Philips, Eindoven, Netherlands) at 10 kV.

### AFM Imaging

A Multimode AFM with Nanoscope IV controller (Bruker Corp, Santa Barbara, CA, USA) was used for all the imaging experiments. The DM tissue sections with and without FG were stored at 4°C prior to imaging. Semicircular portions of tissues with an approximate radius of 0.5 cm were moistened with phosphate buffer saline and mounted on cover slips. Briefly, the samples were air-dried prior to being mounted on the AFM stubs for imaging. Images were captured in tapping mode using a phosphorus doped silicon tips (RTESP; Bruker Corp, Santa Barbara CA, USA) with resonance frequency of ∼250 kHz, and a spring constant of 20–80 N/m. All images were acquired at a scan rate of 0.5 Hz. An area of 10 µm×10 µm was scanned and an average of four reference areas from each sample was used for calculating roughness and skewness values. Height, amplitude and phase images were simultaneously acquired. Three-dimensional images were generated with the software provided by the manufacturer (Nanoscope 6.11 v1, Bruker Corp.).

### Statistical Analysis

The data was reported as mean ± standard error of mean (SEM). Analyses of multiple groups were performed with Mann-Whitney U test to compare between the groups using statistical software, SPSS version 17.0. The level of significance was calculated with *P* value less than 0.05.

## Results

### DM scaffolding and histological analysis

**Figure 3:**
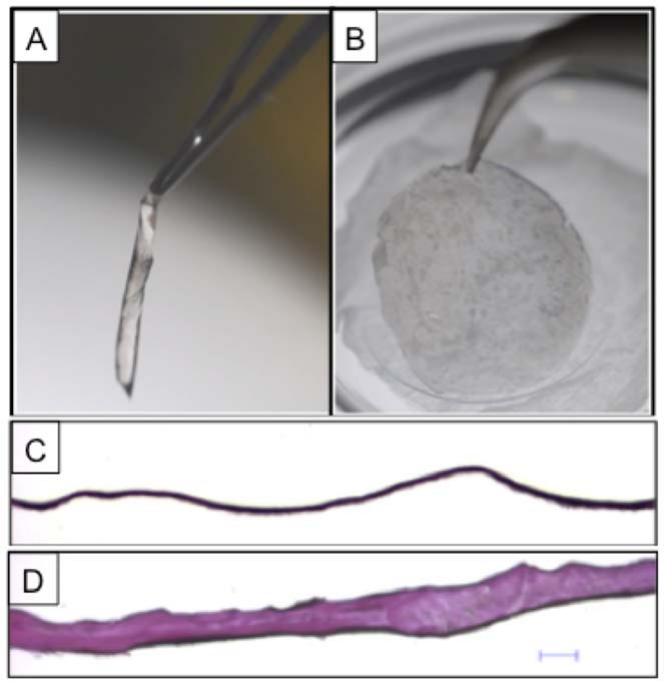
Images of Human Descemet's membrane (DM) before application of fibrin glue (FG), showing the tendency of scrolling (A) and after application of fibrin glue (B), which acts as a support to provide structural rigidity. Hematoxylin and eosin staining images of DM before (C) and after (D) application of fibrin glue, showing differences in measured thickness of the membrane.

The FG sprayed on DM from a distance of 5 cm and a pressure of 10 psi using the EasySpray system distributes a uniform layer of glue over the membrane surface. This treatment provides a temporary scaffold to the tissue, increases the rigidity and prevents it from scrolling over (Fig. 3B), which is an inherent property of human DM under aqueous conditions (Fig. 3A). Histological examination under light microscopy using hematoxylin and eosin staining of sections cut from DM embedded in OCT with FG showed an increase in tissue thickness of ∼50 µm (Fig. 3D) as measured with ImageJ software [35] compared to a native DM (Fig. 3C).

### Fibrin glue application increased the stiffness of DM

**Figure 4:**
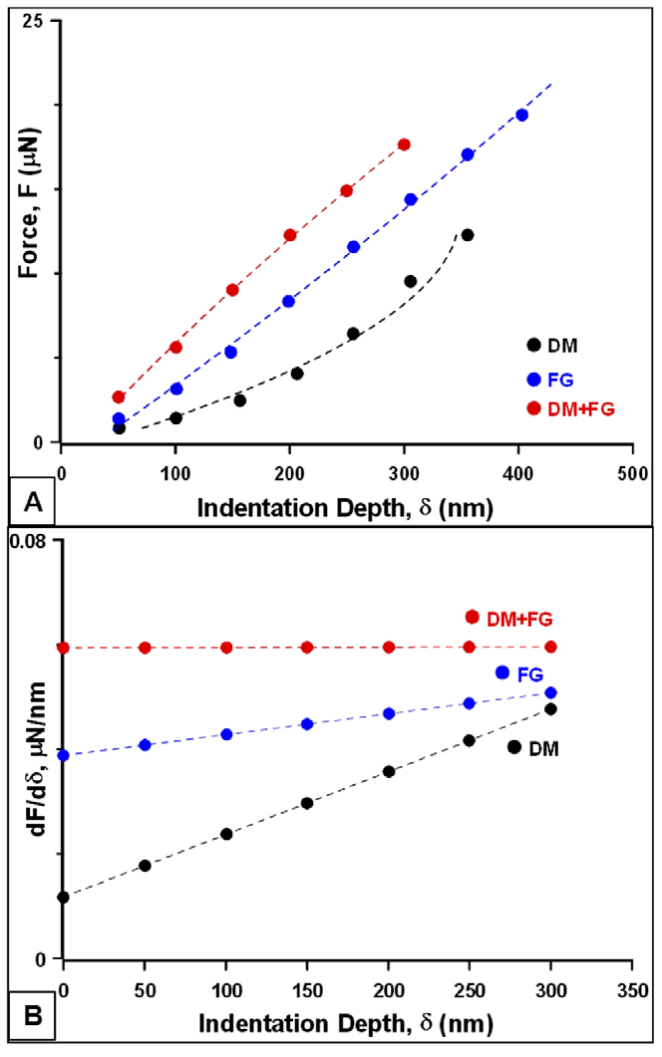
AFM nanoindentation tests were performed on DM with and without fibrin glue (FG) and on a layer of FG sprayed on the PDMS substrate. (A) Plots of force, F (µN) vs. indentation depth, δ (nm). Curves were fitted for data obtained from DM, DM+FG and FG samples. Each curve represents the average obtained from 10–12 curves with the standard deviation being very small. (B) We calculated and plotted the slope dF/dδ vs indentation depth δ. Comparison of the slopes of DM, DM+FG and FG indicated that DM+FG is much stiffer than FG and DM. However, DM displays non-linear behavior as compared to DM+FG and FG as shown by the steep increase in dF/dδ with indentation depth.

Loading force vs. indentation depth curves were obtained by indenting a AFM conical tip on at least 10 different locations on the surface of DM sample. The data obtained for each force curve was plotted from the cantilever deflection (force) and z piezo displacement (indentation depth). The force curves were collected using the straight-line approach, which initially involved minimal interactions of the tip with the surface [36]. There was then a gradual increase in the deflection of the cantilever as the tip comes into contact with the surface, which was represented by the approach curve in the graph and, later as it pulled back to form the retraction curve. The force curves were analyzed for all the three test specimens- DM, DM+FG and FG (Fig. 4A). The equations obtained from fitting the curves were differentiated and plotted against indentation depth (dF/dδ vs. δ) for the analysis of the local slopes (Fig. 4B). This slope gave an indication of the stiffness of the DM, DM+FG and FG, i.e. how much indentation force was needed to result in a unit of indentation depth. The local slope of DM+FG was found to be greater than that of DM and FG (Fig. 4B). However, the increase in the slope values with indentation is much higher in DM when compared to DM+FG and only FG.

### Fibrin glue application increased the relative hysteresis of DM

**Figure 5:**
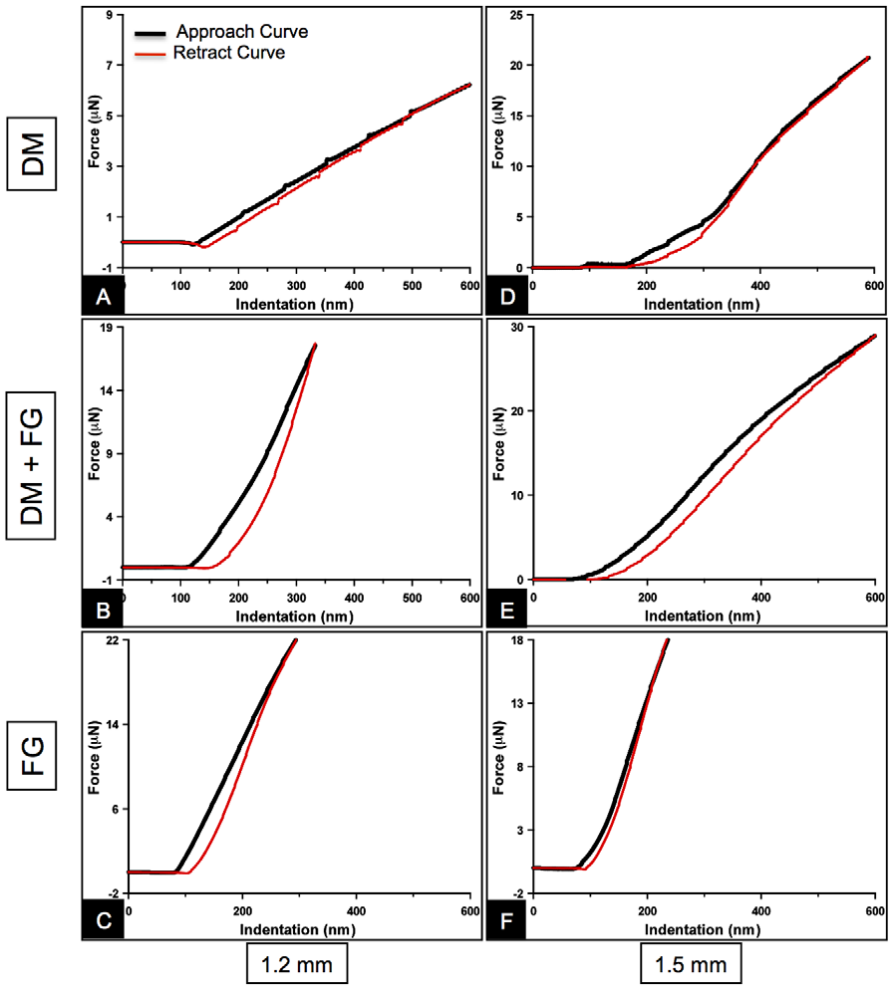
Representative force curves obtained after indenting DM with and without FG suspended on the punched holes of diameter 1.2 mm and 1.5 mm on a PDMS substrate to determine the hysteresis in a sample. The curves were plotted between separation or indentation (δ = z–d) where z is the piezo-displacement and d is the cantilever deflection in x-axis and force applied by the indenter on y-axis for DM (A), DM sprayed with FG (B), and FG alone (C), in the 1.2 mm diameter group. Similar force curves were plotted for DM (D), DM+FG (E), and FG alone (F), obtained from the 1.5 mm diameter group. The black solid line indicates the approach curve when the tip contacts the sample and the red line represents the retract curve when the tip moves away from the sample.

**Figure 6:**
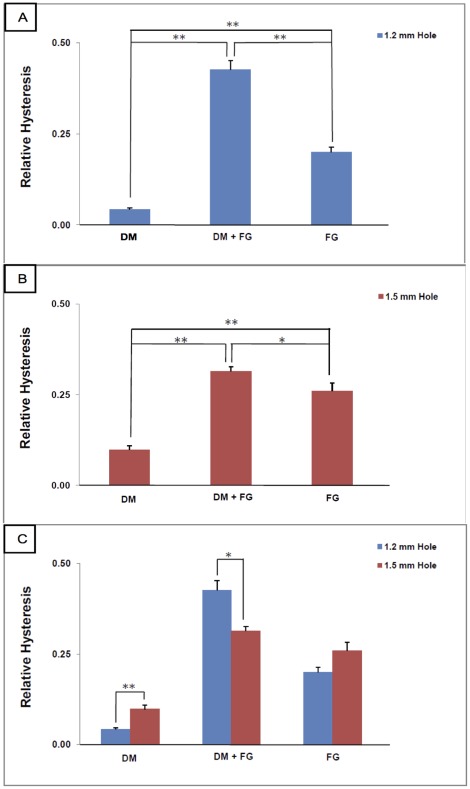
Relative hysteresis measured from the force curves with the area under the approach and retract curves. From these force curves, hysteresis was compared between the samples by calculating the difference in the area of the approach and retract curve by trapezoidal law. Values were compared between DM, DM+FG, and FG alone, indented over 1.2 mm (A) and 1.5 mm diameter holes (B) and between 1.2 and 1.5 mm groups (C). Each data represents the average hysteresis obtained from 10–12 force curves. The error bar represents SEM. * and ** indicate the significant differences at *P*<0.05 and *P*<0.001, respectively.

The force curves (Figure 5) of the FG coated DM displayed considerable hysteresis, i.e., a measure of energy loss during a cycle. Hysteresis values were derived from the area under the approach and the retract curves (using Trapezoidal rule for the area under a curve calculation) by plotting force vs. indentation curves in various experimental conditions as shown in Figure 5A–F. The relative hysteresis measured on the PDMS gel substrate with a punched hole of 1.2 mm exhibited a ∼10-fold increase with a mean of 0.40±0.02 in DM+FG group (*P*<0.001) compared to a mean of 0.043±0.004 obtained in native DM group; a ∼5-fold increase (*P*<0.001) compared to the FG group (mean = 0.20±0.013). The relative hysteresis in native DM group was lower compared to the DM+FG group (*P*<0.001; Fig. 6A). Similar experiments performed on the PDMS gel substrate with a punched hole of 1.5 mm hole showed the same results in that DM+FG (mean = 0.31±0.011) caused a significant increase in relative hysteresis compared to the DM group (mean = 0.097±0.011; *P*<0.001) and FG group (mean = 0.26±0.023; *P*<0.05). The FG group also displayed a greater hysteresis values compared to the native DM group (*P*<0.001; Figure 6B).

Figure 6C depicts the differences in relative hysteresis values obtained between the 1.2 mm and 1.5 mm punched hole groups. There was a significant increase in the force curves derived by indenting on native DM attached to PDMS substrate in 1.5 mm punched hole group compared to the 1.2 mm hole group (*P*<0.001). In contrast, the DM+FG group displayed a significant decrease in mean relative hysteresis values in the 1.5 mm group when compared to 1.2 mm group (*P*<0.05). However, no significant differences were observed in the relative hysteresis values obtained from the FG sprayed PDMS substrate between 1.2 mm and 1.5 mm groups (*P* = 0.073).

### Fibrin glue enhanced the flexural rigidity of DM

**Figure 7:**
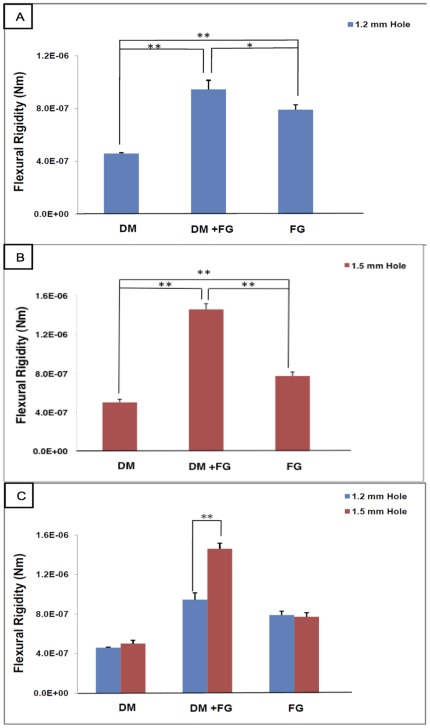
Comparison of flexural rigidity of DM, DM+FG, and FG alone. Flexural rigidity was calculated from the force curves at the indentation depth of 200–500 nm of each sample, using equation 3. Values were obtained from the force curves indented over 1.2 mm (A) and 1.5 mm (B) diameter holes and compared between the two groups (C) at the indentation depth of 200–500 nm. Each data represents the average values from 10–12 force curves. Error bar represents SEM. * and ** represent the significant differences at *P*<0.05 and *P*<0.001, respectively.

The flexural rigidity of DM was calculated based on the elastic deformation of DM bound to a surface containing holes with a typical size of 1.2 mm and 1.5 mm using the conical tip of an AFM. In the group where DM was sprayed with the FG (mean = 9.45±0.32×10^−7^ N/m) and attached on a 1.2 mm hole, the flexural rigidity was shown to be significantly greater than that of the native DM (mean = 4.58±0.06×10^−7^ N/m, *P*<0.001) or FG (mean = 7.9×10^−7^±0.37×10^−7^ N/m, *P*<0.05) groups (Fig. 7A). In addition, a significant difference was found between the native DM versus FG groups (*P*<0.001). In the 1.5 mm group, similar observations were made in the DM+FG group (mean = 1.46±0.058×10^−6^ N/m) where flexural rigidity was significantly higher compared to the native DM (mean = 5.02±0.58×10^−7^ N/m, *P*<0.001) or FG (mean = 7.72±0.42×10^−7^ N/m, *P*<0.001) groups. The native DM group also had a significant decrease in flexural rigidity compared to the FG group alone (*P*<0.001; Fig. 7B).

There was a significant increase in flexural rigidity values attained in the 1.5 mm group compared to the 1.2 mm group for DM and FG (*P*<0.001; Fig. 7C). However, there was no significant difference in the flexural rigidity values obtained between the 1.2 mm and 1.5 mm groups in the DM alone (*P* = 0.1903) and FG alone (*P* = 0.645) groups.

### Surface topography of the DM using SEM and AFM

**Figure 8:**
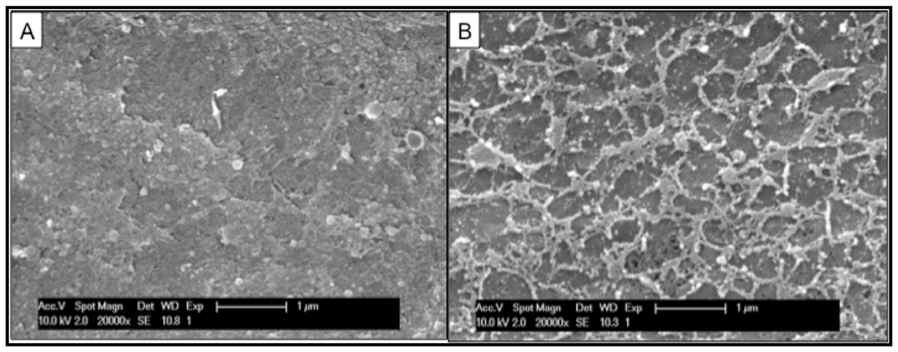
Surface topology of native (A) Descemet's membrane (DM) and DM applied with fibrin glue (FG) (B) using scanning electron microscopy (SEM). Original magnification ×20,000; Bar = 1 µm.

**Figure 9:**
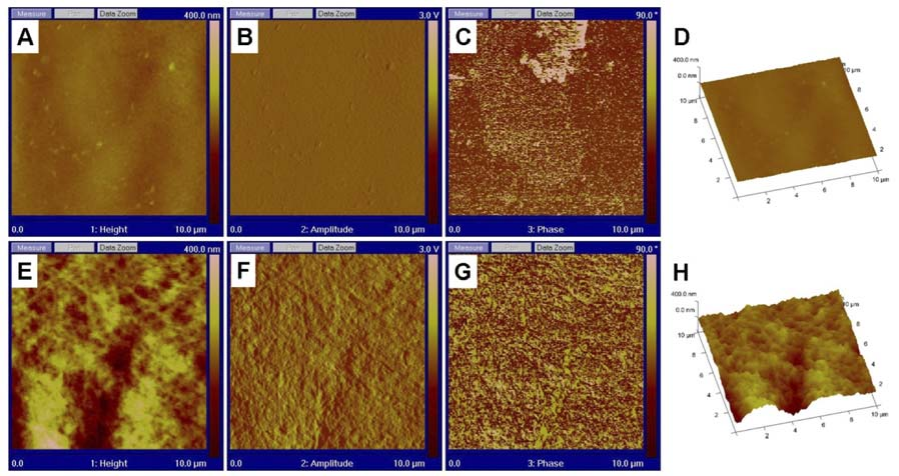
Atomic force microscope (AFM) images showing surface topography of native DM (DM; A–D) and DM with fibrin glue (FG) facing up (E–H). A and E: Height data; B and F: Amplitude data; C and G: Phase data. D and H represent a 3D presentation of topographical map of native DM and DM+FG, respectively. Image scale = 10 µm×10 µm.

The surface topography of native and FG-sprayed human DM was studied using SEM (Fig. 8) and AFM (Fig. 9). In SEM photographs, DM displayed randomly arranged collagen fibrils forming a fine meshwork (Fig. 8A). The addition of FG to the surface of the endothelial side of DM showed a layer of entangled fibrin meshwork (Fig. 8B).

AFM operating in the tapping mode illustrated a smooth and homogenous surface obtained from a native human DM with sub-nanometer lateral resolution when compared to the presence of large, densely packed interwoven fibers of fibrin on the surface of DM/endothelium sprayed with glue. The surface roughness (R_rms_) from the DM sprayed with FG was 33.33±5.99 nm illustrating that the surface of DM was significantly serrated (*P*<0.05) compared to native DM (13.34±1.664 nm). However, the skewness values of the DM (1.64±0.82) showed uneven distribution of roughness data about the mean data profile when compared to DM sprayed with FG (0.15±0.21).

## Discussion

Our study has demonstrated that the application of a uniform layer of FG to native DM (measuring 10–15 µm) increases the elasticity and stiffness of the tissue as measured by AFM nanoindentation. Hysteresis data showed a significant increase in levels of energy loss in DM+FG group. Furthermore, there was an increase in the bending or flexural rigidity of the membrane treated with glue, which in turn increased the stiffness of the DM. Overall, the data suggested that FG sprayed on DM using the EasySpray applicator modified the biomechanical properties and provided a scaffold which increased the rigidity of the tissue, thus preventing it from scrolling, which is a natural tendency of native DM in aqueous conditions.

Basement membranes have been known to play an important role in the differentiation, proliferation and migration of cells [38]–[41]. DM is a specialized basement membrane of endothelial cells in the cornea. Therefore, the mechanical properties of DM are important in the structure and function of endothelial cells, that are actively engaged in fluid transport and protein synthesis across the corneal stroma. The corneal endothelium is responsible for maintaining the desiccation of the stroma by actively removing water [42], [43]. Previous studies have examined the biomechanical properties of selective layers of the cornea and whole corneal tissue using several biophysical approaches/techniques including strip extensiometry and bulge testing [44], [45]. However, these techniques were not suitable for testing smaller tissue samples. Moreover, measurement of biomechanical properties of isolated DM with a single layer of endothelial cells have not been previously studied.

AFM nanoindentation has emerged as one of the most useful technique for determining the biomechanical properties of soft and thin tissue samples, microscopic cells and even biomaterials e.g. polymer nanofibres [46]–[48]. Recently, a few studies on complete corneas have been reported using this approach [36], [49], [50]. Previous studies have provided considerable information on comparing the Young's modulus of different layers of cornea like Bowman's and DM attached to stromal tissue by various indentation techniques [36], [49], [51]. Although, these studies provided a significant understanding about the biophysical property of different layers, it failed to illustrate the true comparison in terms native DM, which is often used in selective tissue transplantation techniques [2]. In this study, we compared the differences in the indentation forces as well as dF/dδ slopes of native DM, DM+FG FG. Firstly, the force required to indent DM+FG was considerably higher at different indentation depths ranging from 50 to 300 nm as compared with native DM and FG group alone. The dF/dδ slope of DM+FG was also higher, which suggested that FG increased the stiffness of the DM when compared to FG and DM alone. One possible reason could be that the fibrin fibers intermingled and cross-linked with DM to create a three-dimensional matrix that might result in the increased stiffness as indicated by the higher slope value. The fibrin networks formed were found to be similar to the structure and mechanism of blood clot formation during coagulation cascade [52]. Also, DM alone showed higher non-linearity by the increase in dF/dδ with indentation depth as compared to DM+FG and FG groups. Again, this supports the notion that FG application to DM increased the stiffness of the tissue when indented by the AFM tip.

For endothelial decompensation, the latest surgical approach is a newly developed technique called DMEK [7], [9]–[11]. It is a “tissue-substitution” procedure where normal corneal thickness is maintained. However, due to the thinness of DM (∼10–15 µm), it has a tendency to curl up or roll upon itself (endothelial cell out) in an aqueous phase without the support of corneal stroma. In the present study, we showed that application of FG increased the strength and overall stiffness of DM. This increase is attributed to higher density membrane fibers formed as a result of FG cross-linking, which provided a scaffold to this very thin and elastic DM.

Most biological membranes are viscoelastic in nature as such some of the energy used in indenting the membrane gets dissipated. This loss can be quantified as the relative hysteresis [37]. In the present study, there was a significant increase in the relative hysteresis in DM attached to the PDMS substrate and sprayed with FG. The presence of an additional layer of FG on the DM increased the relative thickness of the DM and hence increased the viscous energy loss through the indenter.

Flexural or bending rigidity, which is associated with the resistance offered by a biological membrane to bending, was also investigated. The membrane, which was fixed mounted over circular holes of diameters 1.2 mm and 1.5 mm punched on PDMS substrate, was subjected to bending by applying a point load at the center of the membrane using cantilever tip. The flexural rigidity was then calculated using the bending theory for a circular plate [34]. The flexural rigidity of the membrane is dependent on the thickness, elastic property and magnitude of applied load. The flexural rigidity of DM sprayed with FG was significantly higher when compared to both the native DM and FG groups. This is again due to the presence of a layer of FG acting as a scaffold and providing more resistance to flexure or bending when compared to native DM. The results confirmed that even at a higher range of load applied, DM covered with FG would have lesser deflection and hence more resistance to bend, thus yielding higher flexural rigidity.

Surface topographical examination of DM sprayed with FG using SEM revealed a relatively smooth membrane surface with numerous cross-linked fibers intermingled with pores crisscrossing and overlapping with each other, forming a continuous network. The three-dimensional structure, formed as a result of FG application, creates a central porous matrix on the membrane as seen by SEM which resists compression, allows fluid transport and hence restrains the rolling over property of DM. This led to the significant increase in flexural rigidity and stiffness obtained in the present study. AFM imaging revealed higher fractal surface features in the DM treated with FG but a reduced skewness compared to native DM, which increases the relative hysteresis and biomechanical properties of the membrane. Previous study has reported the rheological property of fibrin gels formed from fibrinogen and thrombin. Fibrin gels have high nonlinear elastic behavior, which makes them stiff at higher strain to resist deformation [53].

To conclude, our results showed that the application of FG not only mitigated the inherent property of DM to scroll but also increased the rigidity and with respect to increased hysteresis and flexural rigidity. This was further confirmed by topographical imaging using SEM and AFM, where FG formed a meshwork of fibers on the membrane surface and provided extra support to the ultrathin DM.
